# Diagnostic challenges of hemophagocytic lymphohistiocytosis in child with multiorgan dysfunction in a low-resource setting: A case report

**DOI:** 10.1016/j.amsu.2021.102630

**Published:** 2021-07-30

**Authors:** Maria Christina Noi Sedu, Desy Rusmawatiningtyas, Firdian Makrufardi, Intan Fatah Kumara, Nurnaningsih, Melissa Hines

**Affiliations:** a Department of Child Health, Faculty of Medicine, Public Health and Nursing, Universitas Gadjah Mada/Dr. Sardjito Hospital, Yogyakarta, 55281, Indonesia; b Department of Pediatric Medicine, Division of Critical Care, St. Jude Children's Research Hospital, Memphis, TN, USA

**Keywords:** Hemophagocytic lymphohistiocytosis, Multi-organ dysfunction syndrome, Child, Case report, Low-resource setting

## Abstract

**Introduction:**

and importance: Hemophagocytic lymphohistiocytosis (HLH) is a life-threatening hyperinflammatory syndrome as a result of dysregulation of the immune system. Physicians in the intensive care unit (ICU), especially pediatricians, need to know how to recognize the diagnostic criteria and spectrum of HLH clinical presentations because early detection and timing of initial therapy affect the survival rate of the patient.

**Case presentation:**

A 7-year-old female patient was referred to the pediatric ICU (PICU) at our tertiary hospital because of the suspicion of severe sepsis with shock and disseminated intravascular coagulation. On the fifth day of treatment, the patient was intubated and given a mechanical ventilator after experiencing respiratory failure. On the seventh day in the PICU, high fever persisted and the patient developed worsening acute kidney injury with oliguria. When worsening conditions continued, the patient experienced hypotension and cardiac arrest. The patient died on the 8th day of treatment at PICU due to severe shock and multiorgan failure.

**Clinical discussion:**

HLH complications can be life-threatening with documented ICU mortality of 35%, even with an appropriate initial therapeutic approach. Patients with HLH can have rapid progression of disease and often require a significant amount of ICU supportive care, including vasopressor support, significant amount of blood products, ventilator support, and renal replacement therapy.

**Conclusion:**

The diagnosis of HLH should be considered if there are cases with persistent, prolonged fever, organomegaly, cytopenias and evolving Multi Organ Dysfunction Syndrome. It is important for pediatricians to know the diagnostic criteria and possible clinical presentations of HLH.

## Highlights

• A low-resource setting has its own challenges in handling hemophagocytic lymphohistiocytosis
• Hemophagocytic lymphohistiocytosis is a life-threatening hyperinflammatory syndrome
• The diagnostic criteria and clinical presentations of HLH need to be known by physicians

## 1 Introduction

Hemophagocytic lymphohistiocytosis (HLH), also known as hemophagocytic syndrome, is a life-threatening hyperinflammatory syndrome as a result of dysregulation of the immune system characterized by uncontrolled immune cell activation. HLH is often described as two different forms, primary (genetic) or secondary (acquired). In the familial form (primary) of HLH, genetic defects in the cytolytic activity of natural killer (NK) cells and cytotoxic T lymphocytes can lead to the development of HLH or it may be triggered by other primary immunodeficiencies. In pediatric cases, secondary HLH is often triggered by an infectious disease process, but can also be associated with several diseases that stimulate immunological reactions such as malignancy, metabolic diseases, and autoimmune diseases [1]. Provision of early adequate therapy can prevent the occurrence of a “cytokine storm”, so that multi-system organ failure (MOF) and death can be prevented [2].

Until now the diagnosis of HLH is still difficult to establish, especially in the early stages when only a partial picture of the disease appears. Clinical manifestations of HLH can resemble sepsis, Multi Organ Dysfunction Syndrome (MODS), or MODS related to sepsis [2]. Generally, sepsis and MODS are the most common diagnoses and the most common cause of death in a pediatric intensive care unit (PICU). The diagnostic criteria and spectrum of HLH clinical presentations need to be known by physicians who work in the ICU because early detection and timing of initiation therapy affect the survival rate of the patient [[3], [4], [5]]. In Indonesia, some examinations cannot be carried out due to limited resources, thus becomes a challenge in establishing the diagnosis. This study to be the first case report that describes the challenge of HLH diagnosis in Indonesia's low-resource hospital. This case was reported in line with the Surgical CAse REport (SCARE) criteria [6].

## 2 Case presentation

A 7-year-old female patient, previously in good health, came to the regional hospital with complaints lasting over 4 days of fever, vomiting, myalgia, headaches, and the emergence of multiple ecchymoses in the extremities without a history of trauma. No previous travel history and other family members in good condition. Laboratory results showed pancytopenia with anemia, low leukopenia (900 cells/ml), severe thrombocytopenia (19,000 cells/ml), and low absolute neutrophil count (ANC). Non-structural protein 1 (NS1) and serological tests for Dengue and Salmonella were negative. Urine culture was negative. Because of the suspicion of severe sepsis with shock, and disseminated intravascular coagulation (DIC) this patient was referred to Dr. Sardjito Hospital. Upon admission to the PICU, the patient was given fluid resuscitation and an inotropic agent (dobutamine 7.5 mcg/kgBW/min) for treatment of shock. The patient's general condition looked pale with mild jaundice. Patient was febrile with (39.5 degree Celsius) normotension (100/55 mmHg), but tachycardic (150 kpm). The physical examination found edema of both limbs, abdominal distension, and hepatosplenomegaly. Patient had notable ecchymosis in the puncture area and coffee ground gastric contents from emesis into the nasogastric tube (NGT). Initially, the examinations of the respiratory system, and neurology were within normal limits.

Complete blood tests (Table 1) showed anemia, leukopenia, neutropenia, and thrombocytopenia. Liver function tests revealed increasing levels of transaminases, conjugated hyperbilirubinemia, hypoalbuminemia, and hypertriglyceridemia (936 mg/dL). The coagulation profile showed the prolongation of activated partial thromboplastin time (aPTT) and partial thromboplastin time (PPT) with hypofibrinogenemia (<8 mg/dL). Blood ammonia levels were increased with ferritin levels >6000 ng/ml and high procalcitonin. Analysis of kidney function was within normal limits. Hepatitis A, B, and C serology results were negative. The abdominal ultrasound examination found hepatosplenomegaly and ascites.

**Table 1:** Laboratory examination results during treatment.

	Days of stay							Unit	Reference range
	1	2	4	5	6	7	8		
RBC	2.73	2.86	1.37	2.15	41	3.41	2.52	10∧6/μL	4.00–5.20
Hemoglobin	7.1	7.8	3.9	6.3	11.6	9.6	7.2	g/dL	10.2–15.2
Hematocrit	20.4	22.1	11	17.6	34.8	30.1	22.8	%	34.0–48.0
WBC	1.36	1.53	2.18	3.33	2.39	1.68	0.91	10∧3/μL	5.00–17.00
Neutrophil	22.7	35.3	55.5	72.7	59.5		37.4	%	30.0–60.0
Lymphocyte	72.1	60.1	31.7	16.8	23.8		53.8	%	29.0–65.0
Monocyte	3.7	3.3	11.9	10.5	14.6		8.8	%	2.0–11.0
Eosinophil	0	0	0	0	0		0	%	1.0–4.0
Basophil	1.5	1,3	0.9	0	2.1		0	%	0.0–2.0
ANC	0.31	0.54	1.21	2.42	1.42		0.34	10∧3/μL	1.50–11.00
Platelet	132	22	30	29	34	16	19	10∧3/μL	150–450
Ferritin				>6000,00			>6000.00	ng/mL	20.00–250.00
AST	5772			1950			2375	U/L	<52
ALT	861			391			377	U/L	<78
Total Bilirubin				5.01		10.04		mg/dL	<1.00
Conjugated Bilirubin				3.83		9.25		mg/dL	0.00–0.20
Unconjugated Bilirubin				1.18				mg/dL	<0,40
Ammonia (NH3)				107.9				μL/dL	18.7–86.9
Albumin	2.15	2.61	3.33	3.71		3.47		g/dL	3.40–5.00
PPT		46.5		19.2	16.7	23.3	28.7	second	14.0–15.8
aPTT		74.8		42	40.9	57.6	55.6	second	31.4–40.8
Fibrinogen		<8		88		135	300	mg/dL	148–380
D-dimer		3644		6449		339	6831	ng/mL	<230
BUN	6.1			16.4		30.6	56.1	mg/dL	5.0–18.0
Creatinine	0.18			0.37		0.48	2.1	mg/dL	0.60–1.00
Procalcitonin	11.61			2.68				ng/mL	<0.50

RBC: red blood cell, WBC: white blood cell, ANC: absolute neutrophil count, AST: alanine aminotransferase, ALT: aspartate aminotransferase, PPT: partial thromboplastin time, aPTT: activated partial thromboplastin time, BUN: blood urea nitrogen.

Patient was given transfusions of packed red blood cells (PRC), platelets, cryoprecipitate, and fresh frozen plasma (FFP). Additionally, the patient was given broad-spectrum empirical antibiotic therapy for sepsis after blood cultures were done.

On the fifth day of treatment at the PICU, after she experienced respiratory failure, the patient was then intubated and given a mechanical ventilator. Blood gas analysis revealed respiratory acidosis. Chest X-rays showed pulmonary bleeding. The patient further had decompensation with hypotension and was given fluid resuscitation and additional inotropic agents and vasopressors (dobutamine 10 mcg/kg/min, norepinephrine 0.3 mcg/kg/min, epinephrine 0.3 mcg/kg/min) and glucocorticoids (hydrocortisone 2 mg/kgBW followed by a maintenance dose of 50 mg/m^2^/24 hours) due to suspicion of catecholamine resistance shock. On the seventh day of treatment in PICU, high fever persisted and the patient developed worsening acute kidney injury with oliguria. Laboratory evaluation results revealed anemia, leukopenia, neutropenia, and thrombocytopenia. Peripheral blood bacterial culture obtained negative results. The Pediatric Logistic Organ Dysfunction (PELOD) score was 17. Other laboratory analysis showed that creatinine was increased (2.1 mg/dL) with a decreasing in glomerular filtration rate (GFR) (25.17 ml/min/1.73 m^2^). Patient was given furosemide (0.5 mg/kg/hour) and transfusions of PRC, platelets, FFP, and cryoprecipitate. Immunosuppressive therapy with dexamethasone were given at a dose of 10 mg/m^2^/24 hours intravenously. However, worsening conditions continued and the patient experienced hypotension and cardiac arrest. The patient died on the 8th day of treatment in the PICU due to severe shock and multiorgan failure.

Patient was diagnosed with HLH based on the HLH criteria, which identified 5 of 8 HLH-2004 diagnostic criteria with confirmation based on HScore >169. Examinations of genetic studies, bone marrow biopsy, and NK and CD25 cell activity, were not available.

## 3 Discussion

Here, we reported a case of a 7-year-old female patient with HLH and multiorgan dysfunction in our low-resource setting hospital. The diagnosis of HLH is based on the criterion of HLH-2004 (Table 2). Five of the 8 criteria must be met to establish a diagnosis of HLH, but diagnosing is often a challenge because not all diagnostic criteria can be fulfilled at the same time [8]. In the case of our patient, 5 criteria were found, which are consistent with the diagnosis of HLH, namely: remittent fever, splenomegaly, pancytopenia, hypertriglyceridemia, hypofibrinogenemia and hyperferritinemia. Bone marrow aspiration was not done due to clinical instability, also Interluekin-2 (IL-2) receptor test and NK cell activity could not be done because they are not available in our hospital. According to some authors, these two paraclinical examinations are not widely available in most hospitals and require a long time to get results which will delay the diagnosis of HLH [8].

**Table 2:** Diagnosis criteria of HLH-2004 [8].

Diagnosis of HLH can be established if either A or B is fulfilled
**A. Molecular diagnosis consistent with HLH**
**B. Diagnostic criteria for HLH fulfilled (five of the eight criteria below)** **1. Persistent fever** **2. Splenomegaly** **3. Cytopenias (affecting ≥ two of three lineages in the peripheral blood):** **Hemoglobin <9 g/dL (in infants <4 weeks: hemoglobin <10 g/dL)** **Platelets <100.000/mcL** **Neutrophils <1.000/mcL** **4. Hypertriglyceridemia and/or hypofibrinogenemia:** **Fasting triglycerides ≥265 mg/dL** **Fibrinogen ≤150 mg/dL** **5. Hemophagocytosis in bone marrow or spleen or lymph nodes No evidence of malignancy** **6. Low or absent NK cell activity** **7. Ferritin ≥500 ng/mL** **8. Soluble IL-2 receptor ≥2400 U/mL**
**† Modified with permission from Henter et al.** **HLH, hemophagocytic lymphohistiocytosis; NK, natural killer.**

In a recent adult study, meeting 4 or more criteria was more sensitive and considered almost as specific for the diagnosis of HLH in adult patients than meeting 5 or more. As patients met more criteria, the specificity also increased. Fardet et al. recommended a diagnosis of HLH based on HScore (Table 3), which is based on nine clinical criteria. This HScore was originally used for adult patients with reactive HLH and has had limited validation in the pediatric cohort research [9].

**Table 3:** Diagnosis of HLH based on Hscore [9].

Parameter	No. of points (criteria for scoring)
**Known underlying immunosuppression**^a^	0 (no) or 18 (yes)
**Temperature (°C)**	0 (<38.4), 33 (38.4–39.4), or 49 (>39.4)
**Organomegaly**	0 (no), 23 (hepatomegaly or splenomegaly), or 38 (hepatomegaly and splenomegaly)
**No. of cytopenias**^b^	0 (1 lineage), 24 (2 lineages), or 34 (3 lineages)
**Ferritin (ng/ml)**	0 (<2000), 35 (2000–6000), or 50 (>6000)
**Triglyceride (mmoles/liter)**	0 (<1.5), 44 (1.5–4), or 64 (>4)
**Fibrinogen (gm/liter)** **Serum glutamic oxaloacetic transaminase (IU/liter)**	0 (>2.5) or 30 (<2.5) 0 (<30) or 19 (>30)
**Hemophagocytosis features on bone marrow aspirate**	0 (no) or 35 (yes)

a Human immunodeficiency virus positive or receiving long-term immunosuppressive therapy (i.e., glucocorticoids, cyclosporine, azathioprine).

b Defined as a hemoglobin level of 9.2 gm/dl and/or a leukocyte count of 5000/mm^3^ and/or a platelet count of 110,000/mm^3^.

Hyperinflammation is a common phenotype seen within the ICU, including systemic inflammatory response syndrome (SIRS), sepsis, and other cytokine storm syndromes, such as HLH. All represent hyperinflammation on a spectrum of severity, and in the cases involving patients of HLH and other types of MODS, the severe immune dysfunction leads to the progression of the pathophysiology and the range of conditions is described in the phenotypes. Due to this wide spectrum, there is overlap in the clinical presentations of patients with SIRS, sepsis, resultant MODS and HLH. The incidence of MODS in the PICU ranges from 11 to 27% [10,11]. Suspicion for possible HLH in a patient presenting with sepsis and/or MODS should be heightened in those cases with persistent fever, presence of organomegaly (hepatomegaly and/or splenomegaly, cytopenia (often thrombocytopenia will appear first, then other cell lines will be affected), presence of transaminitis with hyperbilirubinemia, or hypofibrinogenemia. Low fibrinogen, if present, is particularly helpful for HLH diagnosis since patients with sepsis typically present with hyperfibrinogenemia. Presence of hyperferritinemia is also helpful for diagnosis, particularly if extremely elevated >2000 ng/ml (sensitivity 70% and specificity 68%) with specificity rising to 96% in those with ferritin >10,000 ng/mL. Patients with HLH can have rapid progression of disease and often require a significant amount of ICU supportive care, including vasopressor support, significant amount of blood products, ventilator support, and renal replacement therapy. HLH complications can be life-threatening with documented ICU mortality of 35%, even with an appropriate initial therapeutic approach. Pancytopenia and DIC can occur rapidly, followed by MOF in HLH patients. Based on the HLH-94 and HLH-2004 studies, more than 10% of patients die within 2 months of being diagnosed with HLH due to organ bleeding, infection due to neutropenia, or MOF [7]. A study in the United States involving 73 patients with HLH described sepsis and multiorgan dysfunction as the 2 most common causes of death, with survival rates at 1 year only around 48% [12,13]. Another study noted intracranial, gastrointestinal, and pulmonary hemorrhage in one third of patients with poor outcomes. These bleeding complications are described as being directly related to severe thrombocytopenia and hypofibrinogenemia; therefore, aggressive replacement of blood products is needed [14]. Similarly, our patient had gastrointestinal bleeding and severe pulmonary bleeding, due to pancytopenia, hypofibrinogenemia, DIC and died from MOF. Without specific treatment, the outcomes of HLH can be multi-organ failure and ultimately, death. Additionally, central nervous system (CNS) symptoms (e.g. seizures, ataxia, cranial nerve paralysis, hemiparalysis, change in mental status) are unique manifestations of HLH. Patients can have CNS involvement of HLH even without clinical symptoms, but can be accurately diagnosed with brain scans, including computerized tomography (CT) and magnetic resonance imaging (MRI) findings and/or cerebrospinal fluid (CSF) pleocytosis, CSF hemophagocytosis, or increased CSF protein.

Given the quick progression of this disease, early initiation of therapy is imperative. Therapy can be considered with patients that meet 4 of 8 HLH-2004 criteria, especially in patients with rapid deterioration, those where there is high clinical suspicion and those with CNS symptoms confirmed with MRI [8]. As seen in our patient, HLH can be life-threatening, particularly if no specific initial immunosuppressive therapy is given. Our patient had experienced MODS at the time of the HLH diagnosis and was already in a severe clinical condition when referred to our PICU [10].

Based on the results of the HLH-2004 study (no change in outcome with addition of upfront cyclosporine) and the recommendations by Ehl et al., in 2018, the HLH-94 protocol remains the standard of care for HLH-directed therapy [15]. The HLH-94 protocol was written to aid in discerning primary and secondary HLH by weaning therapy within the first 8 weeks of therapy. Prior to the availability of genetic testing, those that have reactivation of disease with weaning of therapy per the protocol were deemed likely to have familial disease and were treated for reactivation and recommended to have hematopoietic stem cell transplantation (HSCT) for definitive therapy. Those with no reactivation were presumed to have secondary disease with close follow-up of therapy. The HLH-94 treatment protocol consists of initiation therapy (8 weeks) and follow-up therapy (after 8 weeks of therapy, until HSCT can be performed). According to the protocol, treatment includes: etoposide (150 mg/m^2^ intravenously, 2 times a week for the first 2 weeks, then once a week for 6 weeks, then every 2 weeks while therapy is continued), and dexamethasone (10 mg/m^2^ for 2 weeks, 5 mg/m^2^ for 2 weeks, 2.5 mg/m^2^ for 2 weeks, 1.25 g/m^2^ for 1 week, and lowered slowly then stopped for 8 weeks and given high doses every 2 weeks with 10 mg/m^2^ for 3 days in advanced therapy). Intravenous immunoglobulin is given every month for the entire therapy period and patients should have pneumocystis jiroveci pneumonia (PJP) and antifungal prophylaxis. Intrathecal therapy is recommended (methotrexate and hydrocortisone) with any evidence of neurological involvement (clinical symptoms; CSF findings, MRI/CT scan findings; weekly therapy between week 3 and week 6) [[16], [17], [18]]. The HLH-2004 therapeutic protocol has shown considerable success with a 5-year survival rate of 66% [19]. However, not all patients will require etoposide and may respond to steroids and treatment of the underlying trigger alone, particularly patients with suspected secondary HLH, with close monitoring (every 12 hours) for lack of response or clinical worsening with rapid addition of etoposide as needed. It is also important to note that for both primary and secondary HLH, the broad consideration and treatment of a possible trigger (e.g. infection, malignancy, etc.) are imperative for the patient to have an adequate response to therapy and ultimately, increased chance of survival.

## 4 Conclusions

The diagnosis of HLH should be considered if there are cases involving persistent, prolonged fever, organomegaly, cytopenias and evolving Multi Organ Dysfunction Syndrome. It is important for pediatricians and especially pediatric intensive care experts to understand how to recognize the diagnostic criteria and possible clinical presentations of hemophagocytic lymphohistiocytosis and initiation of appropriate therapy can be started immediately.

## Declaration of competing interest

No potential conflict of interest relevant to this article was reported.

## Registration of research studies

This is not a ‘first in humans’ report, so it is not in need of registration.
